# Neural mechanisms of confidence propagation in hierarchical partially observable decision-making

**DOI:** 10.1016/j.isci.2025.112782

**Published:** 2025-05-29

**Authors:** Risa Katayama, Wako Yoshida, Ken-ichi Amemori, Shin Ishii

**Affiliations:** 1 Graduate School of Informatics, Kyoto University, Kyoto 606-8501, Japan; 2 Department of AI-Brain Integration, Advanced Telecommunications Research Institute International, Kyoto 619-0288, Japan; 3 Centre for Integrative Neuroimaging, FMRIB, Nuffield Department of Clinical Neuroscience, University of Oxford, OX3 9DU Oxford, UK; 4 Department of Neural Computation for Decision-making, Advanced Telecommunications Research Institute International, Kyoto 619-0288, Japan; 5 Institute for the Advanced Study of Human Biology, Kyoto University, Kyoto 606-8501, Japan; 6 International Research Center for Neurointelligence, the University of Tokyo, Tokyo 113-0033, Japan

**Keywords:** neuroscience, cognitive neuroscience

## Abstract

Our daily decision-making often occurs in environments that are partially observable and hierarchically organized, introducing multiple sources of uncertainty in the decision-making process. To investigate how the brain incorporates subjective confidence stemming from the hierarchical partially observable structure, we developed the twenty-one task, a “blind” blackjack card game with hidden card-deck types. Combining computational modeling and neuroimaging demonstrates that the ventral insular cortex (vIC), presupplementary motor area (SMA), and dorsal anterior cingulate cortex (dACC) encode confidence in deck-type inference during decision-making. Furthermore, individuals vary in how they modulate value-belief and decision confidence based on their deck confidence; these modulations are predicted by the effect of deck confidence on functional connectivity between the vIC and pregenual cingulate cortex, and between the preSMA/dACC and dorsomedial prefrontal cortex, respectively. Our findings suggest that in hierarchical partially observable environments, confidence for higher-order belief affects lower-order confidence through multiple pathways.

## Highlights

• Higher-order confidence guides behaviors under hierarchical partial-observability
• Higher-order confidence shapes both value-belief and decision confidence
• vIC and preSMA/dACC encode higher-order confidence during decision-making
• Prefrontal-insular connectivity reflects higher-order confidence modulation

## Graphical abstract

**Figure:**
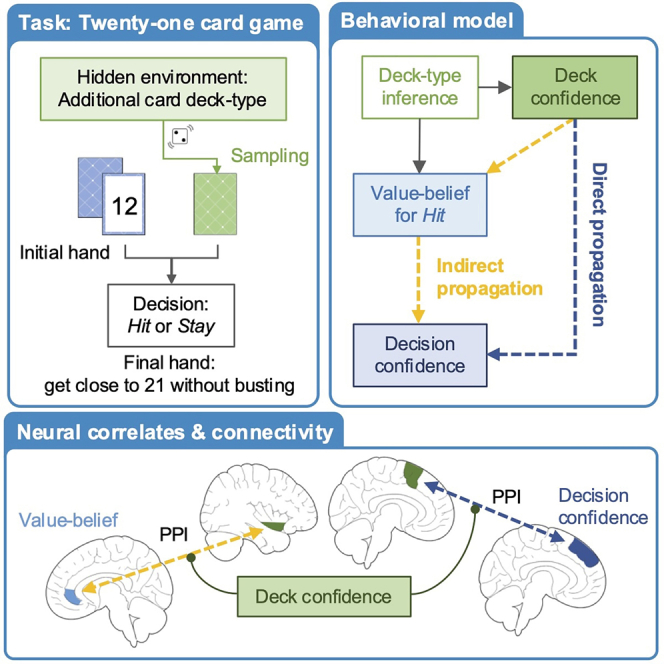

## Introduction

Everyday decisions often take place in environments where only part of the full state is observable. These environments are frequently hierarchical, involving multiple hidden states and additional layers of uncertainty at different levels. To deal with this complexity, decision-makers form beliefs—probabilistic representation of these hidden factors. By using predictive models that address both uncertainty and hierarchy, they can make confident decisions even when faced with incomplete or noisy information.

Consider a scenario where you are ordering lunch at a restaurant chain. You can see the available main dishes in advance. You also have the option to order a lunch set that includes a dessert, though you won’t know which dessert you will receive until after order. If you end up with your least favorite dessert, your satisfaction may drop, and you might regret paying extra for the set menu. However, you know from past experience that the dessert varies by restaurant location—some branches serve your preferred options more frequently than others. Now, at a newly visited restaurant, you must decide: should you order the lunch set or just the main dish? This decision depends on your prediction of what dessert might be included. At the same time, this prediction will depend on your estimation of the trends in the desserts served at this restaurant and your confidence in that estimation. If you are highly confident that this restaurant will serve desserts you enjoy, you might confidently choose the lunch set. Conversely, if you are uncertain about the restaurant’s dessert tendencies, your confidence in ordering the lunch set itself decreases. In this way, your higher-order belief confidence—your confidence in estimating the restaurant’s dessert distribution—shapes both your decision and the confidence you place in it.

In neuroscience, decision-making in hierarchical partially observable environments is often studied in the context of perceptual^1^^,^^2^^,^^3^ and reward learning tasks,^4^^,^^5^ typically formulated using a hierarchical Bayesian model. Previous studies have demonstrated that both humans and non-human primates can track binary contextual variables in partially observable environments based on noisy observations.^5^^,^^6^^,^^7^^,^^8^ Furthermore, recent studies in computational psychiatry suggest that the imprecise weighting of higher-order (i.e., prior) beliefs within a hierarchical information processing is linked to psychotic symptoms such as delusions and hallucinations.^9^^,^^10^^,^^11^^,^^12^^,^^13^ This evidence indicates the interplay between uncertainty in higher-order/upstream information, such as past experiences and contextual inferences, and lower-order/downstream processes, including perception, prediction, and decision-making. However, it remains unclear how subjective measures of uncertainty, or confidence, stemming from different layers interact in the hierarchical partially observable structure, and how the neural mechanisms governing such interactions are implemented.

The successes of hierarchical Bayesian approaches suggest a cascade effect of higher-order state uncertainty influencing downstream processes. However, previous studies have primarily examined uncertainty in higher-order states, such as contextual variables and environmental volatility, within model-based frameworks.^6^^,^^14^^,^^15^^,^^16^ These studies have yet to clarify how subjective confidence specifically contributes to these hierarchical processes. Recent psychonomic studies have highlighted the importance of subjective confidence in decision-making, shifting focus away from purely objective measures like stimulus uncertainty and reward prediction errors.^17^^,^^18^ However, they have not incorporated the hierarchical nature of the environment, leaving a critical gap in understanding how confidence operates across different levels of inference. Furthermore, prior studies often conflated the reliability (or uncertainty) of sensory observations with the uncertainty in contextual inferences and the difficulty of decision-making tied to those inferences.^5^^,^^8^^,^^19^ This overlap makes it difficult to dissociate higher-order confidence (i.e., contextual inference) from lower-order confidence, which pertains directly to the decision itself.

**Figure 1:**
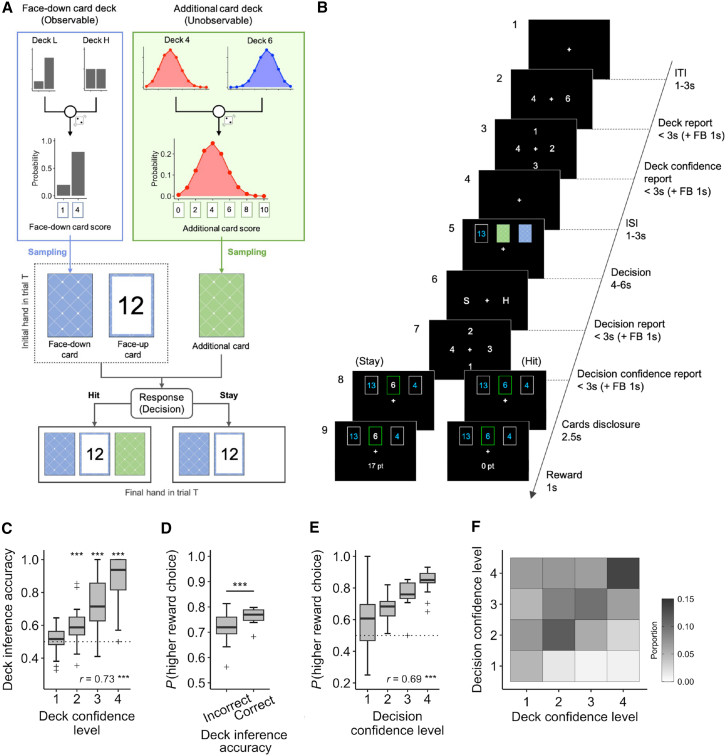
Overview of the twenty-one task, experimental design, and behavioral results (A) In each trial, participants were dealt two cards (blue; face-up card and face-down card) as their hand, after which they were required to decide whether to draw an additional (green) card (hit) or not (stay), with the aim of maximizing the total score of their hand without exceeding 21 points (busting). For each game, which consisted of 16 trials, two types of deck conditions were pseudo-randomly determined. The first condition is the type of face-down card deck, which is indicated to the participants at the beginning of each game. The score of a face-down card is either 1 or 4, with higher-score cards (4) being presented more often than lower-score cards (1) in one deck (80/20%; deck L) and evenly in the other (50/50%; deck H). Another condition is the distribution of additional card decks, which was unknown to the participants. The scores of the additional cards ranged from 1 to 10, with one deck having a distribution biased toward low scores (red; deck 4), and the other toward high scores (blue; deck 6). In the example, the current game condition is deck 4 for the additional card and deck L for the face-down card. (B) On each trial, participants were first asked to select one of the two options (4 or 6) for their estimate of the type of the additional card deck (step 2), and to rate their confidence level on a scale from 1 (low) to 4 (high) (step 3). Next, three cards were displayed: one face-up, one face-down, and one additional card (step 5), and participants were asked to decide as quickly as possible whether to hit (H) or stay (S) (step 6), followed by a confidence rating in their decision (step 7). Afterward, the face-down card was revealed, followed 0.5 s later by the additional card, for a total of 2.5 s (step 8). Finally, the payoff of the trial based on the total score of the participant’s hand was presented (step 9). In this example, if the participant chose to hit, as shown on the right, the payoff was zero due to a bust, as the total score was 13 + 6+4 = 23, exceeding 21 points. (C) The accuracy of deck inference as a function of deck confidence level on the same trial, and was significantly higher compared to the chance level (Wilcoxon signed-rank test, ∗∗∗*p* < 0.001). (D) The probability of selecting a higher-reward choice, compared between the accuracy of deck inference in the same trial. The difference was tested using Wilcoxon signed-rank test (∗∗∗*p* < 0.001). (E) The probability of selecting a higher-reward choice as a function of decision confidence level. In the boxplots in (C–E), each box extends from the lower to upper quartiles, with a horizontal line at the median, whiskers indicate 1.5×IQR, and cross markers denote outliers. The dashed line signifies chance level. (F) Joint probability matrix illustrating the confidence levels in deck inference and decision-making. Each square within the heatmap represents the proportion of the reported deck confidence level (horizontal axis), followed by the decision confidence level (vertical axis).

To address these questions, we designed a twenty-one task, an economic decision-making task with contextual inference. We incorporate probabilistic observations of context into a blackjack-like reward maximization problem. In this “blind” blackjack-like card game, players can draw a hidden additional card from one of two types of decks, each characterized by a different probability distribution of scorecards (Figure 1A). Thus, even under the same observation, players’ optimal decisions vary depending on the hidden context, specifically, the type of deck currently in play. To maximize rewards while avoiding a hand total exceeding 21, players must expect the score of the additional card which they can draw by identifying the current context based on the accumulated history of additional scorecards revealed in each trial. This task design thus compels player’s decision to incorporate uncertainty, or confidence, stemming from multiple layers of the hierarchical partial-observability: the first- (highest-) order state is which deck type is being used, the second is which scorecard is drawn from the current deck, and the third- (lowest-) order state is what total score of a hand, including face-down cards. By structuring the task around these interdependent levels, the design captures how players integrate confidence across hierarchical layers to guide decision-making in a hierarchical partially observable environment.

## Results

### Behavioral results

Twenty-four healthy participants performed the twenty-one task (Figures 1A and 1B). In each trial, the participants first inferred which of the two types of the additional card deck was in play based on the card score history of previous trials and reported their confidence levels in this inference. They then decided whether to draw a card and add it to their hand (i.e., hit or stay) and reported their confidence in the optimality of their decision (Figure 1B). The accuracy of deck inference significantly improved as the trials progressed within each game (Figure S2C, group-level *r* = 0.79, *p* = 9.3 × 10^−84^; individual-level, *r* = 0.84 ± 0.19, Wilcoxon signed-rank test, *p* = 2.1 × 10^−5^) and was also higher when the participants reported greater confidence in their inference (Figure 1C, group-level, *r* = 0.73, *p* = 2.5 × 10^−17^; individual-level, *r* = 0.87 ± 0.19, *p* = 1.8 × 10^−5^). The participants also earned more points when they correctly identified the additional card deck type compared to incorrect guesses (Figure 1D; Wilcoxson signed-rank test, *p* = 4.0 × 10^−4^). For decision confidence, participants were asked to report whether they believed the outcome of their chosen action would be better than that of the unchosen action. In fact, the probability of choosing a higher-reward action was significantly positively correlated with their reported confidence (Figure 1E, group-level, *r* = 0.69, *p* = 6.8 × 10^−15^; individual-level, *r* = 0.78 ± 0.42, *p* = 3.0 × 10^−5^).

**Figure 2:**
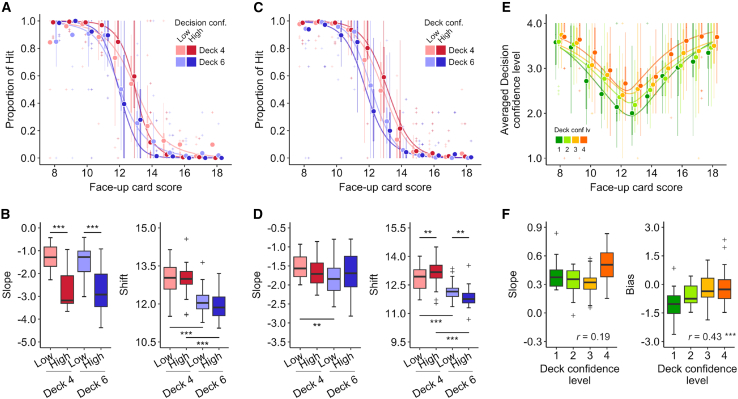
Effect of the deck confidence level on the decision and decision confidence (A) The probability of selecting a hit is shown as a function of the face-up card score with four data conditions, where the data are classified according to the participants’ deck inference (4 or 6) and the decision confidence (high or low based on participant-specific mean split). Each point marker indicates the participant-wise average of the hit probabilities. The solid lines show the mean fits from hierarchical logistic regression with ilogit(P(hit))∼slope×(Faceupcardscore−shift), where ilogit(p) is the inverse logistic function. (B) The magnitude of the slope of the hierarchical logistic fits shown in (A) was significantly higher (i.e., steeper) for trials with high decision confidence than for trials with low decision confidence, regardless of the deck inference (left; Wilcoxon signed-rank test, ∗∗∗*p* < 0.001). The shift (right) was higher on trials in which the participants estimated the current deck was deck 4 than on trials in which they estimated deck 6, regardless of the decision confidence level. (C) Participant-wise probability of choosing hit as a function of the face-up card score, when the data were categorized binarily according to the deck inference and the deck confidence level. The format is the same as in (A). (D) The shift of the hierarchical logistic fits shown in (C) was significantly different depending on both the deck inference and its confidence level (Wilcoxon signed-rank test, ∗∗*p* < 0.01 and ∗∗∗*p* < 0.001). When the participants estimated the additional card deck as deck 4, the shift was higher in the high compared to that in the low-deck-confidence trials. The opposite pattern was observed in the deck 6 trials. The format is the same as in (B). (E) Participant-wise averaged decision confidence levels when the data were divided according to the reported deck confidence level, as a function of the face-up card score. Each point marker indicates the participant-wise average decision confidence. (F) Slope (left) and bias (right) of the hierarchical regressions in (E). The estimated bias value was significantly correlated with the deck confidence level (*r* = 0.52, ∗∗∗*p* < 0.001). In the boxplots, each box extends from the lower to upper quartiles, with a horizontal line at the median, whiskers indicate 1.5×IQR, and cross markers denote outliers. (F) Joint probability matrix illustrating the confidence levels in deck inference and decision-making. Each square within the heatmap represents the proportion of the reported deck confidence level (horizontal axis), followed by the decision confidence level.

In this task, participants evaluated their confidence levels in both deck inference and hit-or-stay decision. The reported levels of these two types of confidence were significantly correlated (Figure 1F, group-level, *r* = 0.30, *p* = 6.4 × 10^−157^; individual-level, *r* = 0.20 ± 0.15, *p* = 3.4 × 10^−5^), yet differed in 40.1 ± 13.7% of the trials. These results led us to ask how these different kinds of confidence influenced participants’ decision behaviors. To explore this, we first performed a hierarchical logistic regression analysis, dividing the data into 2 × 2 classes based on high or low deck inference and decision confidence levels (determined by participant-specific mean splits; Figure 2A). The estimated shifts were significantly affected by deck inference level, but not by decision confidence level, suggesting that participants adjusted their decisions based on subjective priors (deck inference), even when faced with the same observation (face-up card score) (Figure 2B, right; repeated two-way aligned rank transform [ART] ANOVA, effect of deck inference, *F*(1,23) = 107.9, *p* = 3.7 × 10^−10^; effect of decision confidence, *F*(1,23) = 1.3, *p* = 0.27). It was revealed that, on the other hand, the magnitudes of the estimated slope were larger in the high-decision-confidence trials than in the low-decision-confidence trials (Figure 2B left; effect of deck inference, *F*(1,23) = 0.60, *p* = 0.45; effect of decision confidence, *F*(1,23) = 92.7, *p* = 1.6 × 10^−9^), indicating that when participants were more confident in their decisions, they were more sensitive to the value of the options, regardless of the deck inference.

We then analyzed the data by categorizing participants’ deck inference and confidence levels into high or low, using participant-specific mean split (Figure 2C). In contrast to the analysis based on the decision confidence level, the ART ANOVA revealed a significant effect of both deck inference and deck confidence on shift, with a notable interaction between these factors (main effect of deck inference, *F*(1,23) = 88.6, *p* = 2.4 × 10^−7^; main effect of deck confidence, *F*(1,23) = 1.6 × 10^−2^, *p* = 0.90; interaction, *F*(1,23) = 23.1, *p* = 7.5 × 10^−5^; Figure 2D right). Specifically, when the participants inferred the additional card deck as deck 4, the shift estimated from a hierarchical logistic fit (see Figure 2B caption) was higher in the high-deck-confidence trials compared to the low-deck-confidence trials (Wilcoxon signed-rank test, *p* = 7.2 × 10^−3^), whereas when the participants inferred it as deck 6, the shift estimation was lower in the high-deck-confidence trials than in the low-confidence trials (*p* = 1.0 × 10^−3^). These contrasting effects suggest that when the participants had low confidence in their deck inference, they tended to consider not only the inferred deck state but also the other deck state to guide their decisions. There was no significant effect of either deck inference or deck confidence on the estimated magnitudes of shift (Figure 2D left, main effect of deck inference, *F*(1,23) = 3.9, *p* = 6.0 × 10^−2^; main effect of deck confidence, *F*(1,23) = 2.3 × 10^−2^, *p* = 0.88).

Furthermore, participants’ decision-confidence levels reached a minimum when the face-up card score was around 13, a point where the proportion choosing to hit was at around 0.5, and increased monotonically as the proportion of hit (or stay) deviated from 0.5, regardless of the deck confidence levels (Figure 2E). In contrast, the minimum level of decision confidence showed a significant correlation with the deck confidence level (Figure 2F; *r* = 0.43, *p* = 1.3 × 10^−5^). Statistically similar results to those obtained using data from the behavioral experiment (outside the scanner) were obtained when we analyzed the data from the scanning experiment separately (Figure S1). In this task, we introduced different levels of variability in the face-down scorecard (see STAR Methods), aiming to incorporate observational uncertainty to dissociate the uncertainty in deck inference from that in value-belief. However, no significant differences in participants’ choices or decision confidence were observed between the two types of face-down card decks.

### Computational implementation of the hierarchical model of decision-making incorporating deck confidence

**Figure 3:**
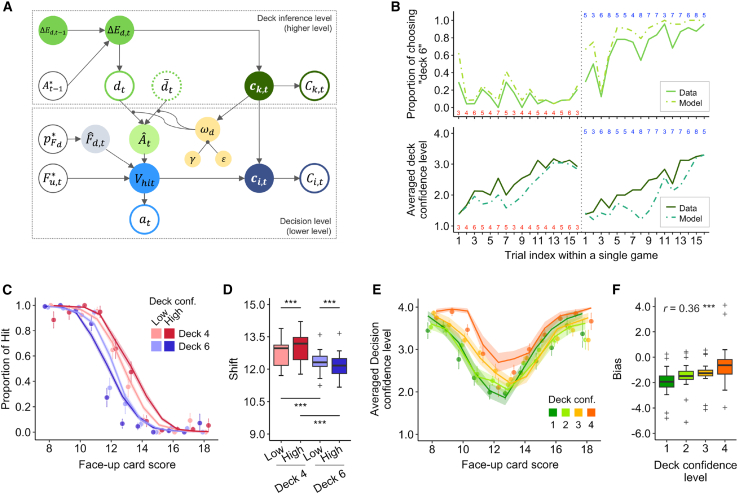
Hierarchical decision-making model and model-based behavioral analysis results (A) Schematic description of the DC-MD model. This model assumes that the participants first update their deck inference (d) and subsequently estimate the value-belief for the “hit” choice ($V_{hit}$) based on the face-up card score ($F_{u,t}^{*}$), the score probability of the face-down card ($p_{F_{d}}^{*}$), and the preceding deck inference. It is hypothesized that the value-belief is dependent on both types of the additional card deck; the inferred deck and the alternative deck type ($\bar{d}$), with the weight ($\omega_{d}$) depending on the deck confidence level ($c_{k}$). The two variables described alongside the edge (gamma, epsilon) are free parameters that regulate the value-belief modulation based on the deck confidence. This model also assumes that the decision confidence level ($c_{i}$) is biased affected by the deck confidence. $C_{k}$ and $C_{i}$ indicate the participants’ reported (i.e., 4-point discretized) deck confidence and decision confidence levels, respectively. The details of the alternatives are described in the STAR Methods. (B) Trial-by-trial behavior of deck inference (top) and deck confidence level (bottom) for the example games. Solid lines show the average choice probabilities and confidence levels of all participants, while dashed lines show the model prediction. The red (or blue) numbers below (or above) the plots indicate the additional card scores in each trial when the (hidden) true deck type was deck 4 (red) or deck 6 (blue). (C) Model-predicted probability of choosing to hit. Solid lines with shaded areas indicate the predictions across the 2 × 2 multiplicative conditions: deck inference (deck 4 or deck 6) × predicted deck confidence (high or low) (mean ± SEM). Point marker indicates the participant-wise average actual hit probabilities, with error bars indicating SEM. (D) Shift of the hierarchical logistic fits based on model-predicted hit probabilities shown in (C). The differences were tested using Wilcoxon signed-rank test (∗∗∗*p* < 0.001). The format is the same as Figure 2B. (E) Model-predicted decision confidence levels. Point markers represent the average actual confidence level reported by participants. (F) Bias of the hierarchical regression of decision confidence in (E). The estimated bias was significantly correlated with the deck confidence level (*r* = 0.35, ∗∗∗*p* < 0.001). The format is the same as Figure 2F.

We developed a hierarchical model of the participants’ decision-making behaviors (Figure 3A). In this model, participants first updated their inference about the type of additional card deck based on the scores of the revealed additional cards from previous trials (Equations 1, 2, 3, and 4). Participants would then estimate the additional card score as a mixture of scores based on both deck types, depending on their deck confidence level (Equations 6 and 7). Specifically, when participants were most confident in their inference, their inferred deck type was weighed using the epsilon parameter (>0.5; Table S1). Conversely, when less confident, the different deck type received more weight by the gamma parameter (Equation 7). They then calculated their *value-beliefs* for the “hit” choice as the difference between the busting threshold, 21, and the expected total score by choosing to hit, i.e., the sum of the score of the face-up card, the estimated score of the additional card, and the expected score of the face-down card (Equation 8). Based on this value-belief, they made their decisions with an increased probability of choosing “hit” when the value-belief was greater. Additionally, their choice accuracy was modulated by the absolute value of their value-belief, with larger values making decisions easier and more accurate (Equation 9). The deck type of the face-down card was predetermined for the participants, allowing its expected score to be calculated uniquely. Our model also assumed that the participants’ decision confidence level was not only shaped by the subjective decision simplicity but also bolstered by the deck confidence, reflecting an upward bias based on perceived deck reliability (Equation 10, see details in STAR Methods).

Our proposed model, termed the deck-confidence-modulated decision (DC-MD) model, accurately replicated the participants’ deck inference and confidence evaluation (deck inference, 87.2 ± 11.0%, Wilcoxon signed-rank test, *p* = 2.1 × 10^−5^; deck confidence, 54.5 ± 12.7%, *p* = 1.8 × 10^−5^), showing a similar evolution to the participants’ actual behaviors within a single game (Figure 3B). Its reproducibility in both hit-or-stay decisions and the corresponding confidence levels was significantly higher than chance (decision, 93.5 ± 3.7%, Wilcoxon signed-rank test, *p* = 1.8 × 10^−5^; decision confidence, 54.4 ± 13.8%, *p* = 2.1 × 10^−5^), successfully capturing key behavioral patterns (Figures 3C–3F). Furthermore, we tested the proposed model against two alternative models (see STAR Methods and Figure S3) and demonstrated that our DC-MD model provided the best explanation for participants’ behaviors (Table S2). Note that, we also evaluated two alternative models for the deck inference phase: the averaging model and the optimal Bayesian model. However, they exhibited lower reproducibility compared with our proposed evidence-based updating model (please refer to Table S2); due to this, we only used the evidence-based updating as a model for the deck inference phase.

### Neural encoding of the components in deck-confidence-modulated decision-making

**Figure 4:**
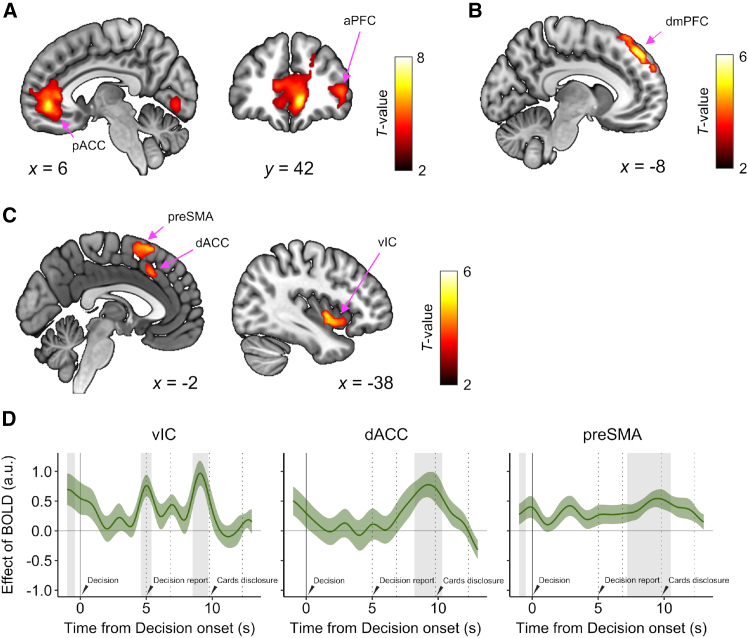
Neural correlates involved in deck-confidence-modulated decision-making (A–C) Neural representation of the chosen value-belief (A), the decision confidence level (B), and the deck confidence level (C) at the onset of the decision-making (step 5 in Figure 1B). The clusters are significant at *p* < 0.05, FWE-corrected, with a cluster-defining threshold of *p* < 0.001, uncorrected. (D) Time course of BOLD signals for the left vIC (left), dACC (center), and preSMA (right) shown in (C). Lines and shaded areas represent the means ± SEM across participants. The gray shaded areas indicate that the effect size for deck confidence (beta weight) was significantly positive (permutation test, *p* < 0.05, FDR-corrected). Vertical dotted lines represent the average onset of events in a single trial (steps 5–9 of the current trial in Figure 1B).

Based on our computational model, we conducted a general linear model (GLM) analysis to identify brain activity related to decision-making by examining two indicators: chosen value-belief and decision confidence, estimated at the decision onset (Figure 1B, Step 5). Activities in the pregenual anterior cingulate cortex (pACC), the right anterior prefrontal cortex (aPFC), the right middle temporal gyrus, the left middle occipital gyrus, the superior temporal gyrus, and the bilateral dorsal insular cortices correlated with the chosen value-belief (Figures 4A and S4B, Table S3). In contrast, activity in the dorsal medial prefrontal cortex (dmPFC) increased as a function of the level of decision confidence (Figure 4B, Table S3).

Next, we examined brain activity related to deck inference and found that BOLD activity in the left posterior insular cortex (PIC) significantly correlated with the deck confidence level at the onset of the deck-type report (Figure S4C). Given the findings from the behavioral and model analyses suggesting that deck confidence influenced subsequent decisions, we further investigated brain activity related to the deck confidence during decision-making. We observed that the left ventral insular cortex (vIC) and the medial part of the prefrontal cortex, specifically within regions overlapping the presupplementary motor area (preSMA) and dorsal anterior cingulate cortex (dACC), showed a positive correlation with the deck confidence levels at the decision onset (Figure 4C, Table S3). These findings imply that these regions encode confidence-modulated signals throughout the decision-making process. We also constructed a GLM incorporating the deck confidence, chosen value-belief, and decision confidence; each of three variables exhibited significant effect in regions similar to shown in Figure 4 (Figure S4D). We then examined the temporal dynamics of these brain activities to understand how each region could contribute to specific cognitive functions. We found that the signal tracking the deck confidence peaked approximately 5 and 9 s after the decision onset in the vIC (Figure 4D left), whereas the dACC signal gradually increased after the decision report onset rather than the decision onset, reaching its peak approximately 9 s after the decision onset (Figure 4D center). The preSMA activity also showed a peak approximately 9 s after the decision onset (Figure 4D right).

### A network underlying deck-confidence-modulated decision-making

**Figure 5:**
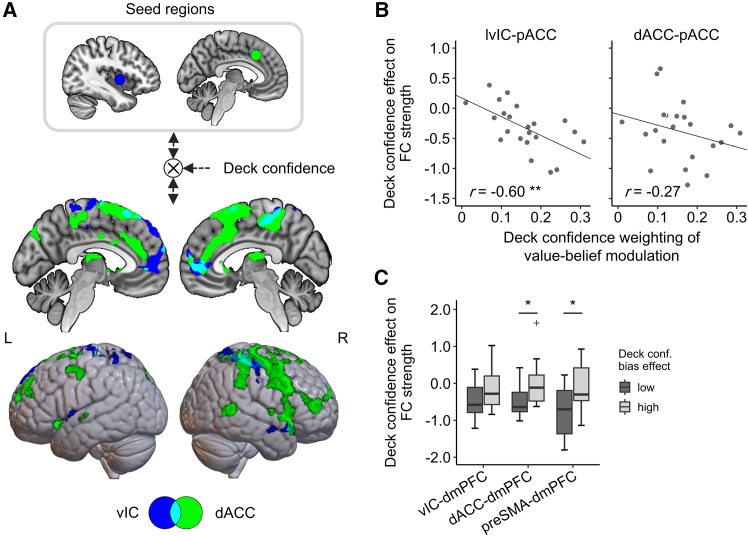
Modulation of functional connectivity between the brain regions engaged in deck-confidence-modulated decision-making (A) Brain regions that show decreases in functional connectivity with the left vIC (blue) and dACC (green) as a function of the deck confidence level during decision-making (for 5 s from the onset of decision). The clusters are significant at *p* < 0.05 (FWE-corrected), with a cluster-defining threshold of *p* < 0.001 (uncorrected). (B) Between-participant correlation between the individual difference in the deck-confidence weighting of value-belief modulation (standardized gamma value) and the modulation of deck confidence on the vIC-pACC functional connectivity (left, *r* = −0.60, *p* = 3.1 × 10^−3^) and the dACC-pACC functional connectivity (right, *r* = −0.27, *p* = 0.22). (C) Effect of deck confidence on the functional coupling strength across the participants with the different levels of the deck confidence bias effect on decision confidence (standardized $\beta_{c_{i}}\left(c_{k}\right)$). The significance of the difference was tested using a one-sided Wilcoxson rank-sum test (∗*p* < 0.05).

The time-series analyses revealed distinct temporal dynamics in the vIC and preSMA/dACC regarding the effect of the deck confidence level (Figure 4D). Considering the peak timing and the delay associated with the hemodynamic response function (HRF), we hypothesized that the vIC contributes to the initial stage processes, such as evaluating value-belief, whereas the dACC and preSMA play a role in higher-order metacognitive functions, including assessing decision accuracy. To examine this hypothesis, we conducted a psychophysiological interaction (PPI) analysis to examine how the deck confidence modulates functional connectivity (FC) between the vIC, dACC, or preSMA, and other brain regions during the period of decision-making (step 5 in Figure 1C). We found that as the deck confidence increased, the strength of FC between the vIC and medial prefrontal regions, including the pACC and dmPFC, decreased (Figure 5A blue). These areas overlapped with those encoding the chosen value-belief (Figure 4A; Szymkiewicz-Simpson [SS] coefficient, 0.38). In contrast, the dACC exhibited FC that was modulated by the deck confidence level not only with the anterior cingulate cortex (including the pACC; *SS* = 0.21) but also with the lateral parts of the frontal lobes (Figure 5A green). In the preSMA, the strength of FC with the dlPFC was negatively correlated with deck confidence, similar to the dACC, while there was no negative correlation in the preSMA-pACC connectivity (Figure S4F). Moreover, it was revealed that the negative effect of deck confidence on vIC-pACC connectivity became more pronounced as participants exhibited greater deck-confidence weighting in their value-belief modulation (i.e., the estimated value of gamma in the DC-MD model; Figure 5B left, *r* = −0.60, *p* = 3.1 × 10^−3^); in other words, individuals who relied more on their deck confidence showed a greater decline in the connectivity strength as deck confidence increased. Meanwhile, the PPI effect of deck confidence on the dACC-pACC connectivity was not significantly correlated (Figure 5B right, *r* = −0.27, *p* = 0.22). These findings suggest that the vIC plays crucial roles in modulating value-belief in relation to the varying levels of deck confidence.

On the other hand, if these regions representing the deck confidence are involved in modulating decision confidence, it is hypothesized that the effect of deck confidence on the connectivity with dmPFC, which encodes the decision confidence, would correlate with the individual’s deck confidence bias effect on decision confidence (i.e., the parameter $\beta_{c_{i}}\left(c_{k}\right)$ in the DC-MD model). The correlation between this deck confidence bias effect and the PPI effect of deck confidence on the connectivity between the vIC, dACC, preSMA, and dmPFC was not significant (vIC-dmPFC, *r* = 0.14, *p* = 0.53; dACC-dmPFC, *r* = 0.30, *p* = 0.18; preSMA-dmPFC, *r* = 0.27, *p* = 0.22). Nevertheless, the deck confidence bias effect on the functional coupling of dACC-dmPFC and preSMA-dmPFC was higher in the individuals with a greater bias effect (Figure 5C, Wilcoxon rank-sum test, dACC-dmPFC, *p* = 1.7 × 10^−2^; preSMA-dmPFC, *p* = 3.8 × 10^−2^). This correlation was not significant for the vIC-dmPFC connectivity (*p* = 9.4 × 10^−2^). These results suggest that the dACC and preSMA mediate the decision confidence, rather than engaging in value processing itself.

## Discussion

In this study, we designed a twenty-one game, inspired by blackjack card game, which integrates an inference problem with value-based decision-making in a hierarchical partially observable environment. We investigated how the human brain incorporates higher-order confidence, specifically, confidence in inferring the context (i.e., the additional card-deck type), into lower-order decision-making processes, such as whether to hit or stay, along with the associated confidence evaluation. Behavioral analyses demonstrated several key findings: (1) participants formed value-beliefs depending on their inferred card-deck type; (2) this dependence on deck inference decreased when they had lower confidence in their deck-type inference (Figures 2C and 2D); and (3) decision confidence was influenced not only by the difficulty in the decision but also by the confidence in the deck inference (Figures 2E and 2F). Based on these results, we proposed a generative model of the participants’ behavior in this card game task.

In our proposed game, the confidence level in inferring the higher-order hidden contextual state was used to arbitrate the value-beliefs across multiple contextual states; that is, the environments where additional cards were drawn from either deck 4 or deck 6. We introduced a behavioral model with a hierarchical structure incorporating interlayer modulation, characterized by the subjective confidence in the deck-type inference. Our model, the DC-MD model, showed better reproducibility of participants’ deck inference and decision compared to the hierarchical Bayesian framework (the hierarchical Gaussian filtering [HGF] model; see Table S2). Our DC-MD model also assumed that confidence in the inference of the higher-order hidden contextual state (i.e., additional card deck-type) directly modulates the decision confidence level, and explained the participants’ decision confidence report more accurately compared to the other alternatives that lacked a deck-confidence bias effect (the deck-confidence-independent decision [DC-ID] model; Table vS2). These results allowed us to apply the confidence-driven decision-making model in explaining behavior within hierarchical partially observable environments.

Through model-based imaging analyses, we found that BOLD activity in the left vIC and the dACC was modulated by the confidence level of deck inference. This suggests that these regions are involved in the belief of a higher-order contextual state that influences decision-making. Previous studies using classic stimulus-response conflict tasks have proposed that the dACC serves as a higher-order mechanism within hierarchical structures,^20^^,^^21^^,^^22^^,^^23^ that is, detecting conflicts and allocating cognitive control resources. However, the role of the dACC was primarily limited to specifying control signals based on observable environmental features (e.g., task-relevant features in the Stroop task),^24^ and its function in uncertain or partially observable environments has remained less clear. Emerging evidence from computational neuroscience has highlighted the involvement of the dACC in hidden state inference (i.e., belief) in uncertain environments in humans,^25^^,^^26^ non-human primates,^27^ and rodents.^28^^,^^29^ However, these studies typically examined state beliefs in non-hierarchical frameworks. Our findings extend this understanding, showing that the dACC is involved in higher-order belief confidence within hierarchical frameworks, bridging gaps in previous research and providing a more comprehensive view of its role.

The insular cortex has been thought to bias behaviors by influencing the regions involved in reward-seeking and self-control^30^ and demonstrated that it is critically associated with representation and processing of risk during risky decision-making.^6^^,^^31^^,^^32^^,^^33^ Although the activity observed in the previous studies was often localized at the dorsal anterior insular cortex (dAIC), our current study has found that this region represents the chosen value-belief—also associated with how unlikely the chosen action is to bust (Table S3). While the anterior part of the vIC has been associated with socio-emotional processing,^34^^,^^35^ several recent studies suggested that the vAIC plays a critical role in context-motivated behavior selection. A non-human primate study has demonstrated that microstimulation in the vAIC pronouncedly disrupts approach-avoidance behavior.^36^ A human study also showed that intracranial electrical stimulation in the vAIC reduced risk-taking behaviors by increasing sensitivity to potential losses, whereas stimulation in the dAIC induced the opposite effect.^37^ Additionally, in the context of sensory-motor decision-making, it was suggested that the insular activity is associated with the uncertainty of *a priori*-acquired information of the events.^15^ Our findings support the existence of functional dissociation between the ventral- and dorsal insular cortices and suggest that the vIC is involved in modulation of behaviors as a higher-order effect.

Our FC analyses revealed distinct coupling patterns of the vIC and dACC/preSMA in which the deck confidence level was encoded during decision periods. The vIC was functionally coupled with the pACC, and the strength of this connectivity was correlated with deck confidence. In addition, a negative relationship between deck confidence and connectivity strength was observed, which aligned with individual differences in the influence of deck confidence on value-belief modulation (Figure 5B). Functional coupling between the vIC, especially its anterior part, and the pACC has been demonstrated in both resting-state^38^^,^^39^ and task-based fMRI analyses,^39^^,^^40^ as well as through diffusion fMRI-based structural connectivity in humans.^41^^,^^42^ The dACC also showed deck-confidence-dependent FC with the pACC, but unlike the vIC-pACC connection, this coupling was not associated with the deck confidence weighting. Notably, the effect of deck confidence on the dACC-dmPFC and preSMA-dmPFC connectivity strength was positively enhanced in participants who demonstrated a stronger bias of deck confidence on decision confidence level (Figure 5C). Based on these observations, we propose two distinct pathways by which higher-order belief confidence modulates lower-order decision confidence in hierarchical partially observable environments: an indirect pathway involving the vIC-pACC coupling for value-belief modulation, and a direct pathway involving the dACC- and preSMA-dmPFC coupling transmits higher-order confidence biases.

In summary, this study demonstrated that in value-based decision-making incorporating hierarchical partially observability, confidence in higher-order belief (i.e., inference about additional card deck) modulates lower-order value-beliefs and exerts influence on decision confidence both directly and indirectly. Imaging and connectivity analyses further implicated the insular-prefrontal network is involved in such hierarchical confidence propagation during decision-making. These findings provide insights into a multi-level cognitive and metacognitive architectures under hierarchically uncertain environments and may contribute to understanding the mechanisms of psychotic symptoms that would emerge from impairments in such hierarchical information.

### Limitations of the study

This study has several limitations that should be acknowledged. First, the effect of the uncertainty of value-belief, which is the intermediate state between the deck inference and decision-making, was not comprehensively examined. Future research should aim to disentangle the contributions of prior-uncertainty from those of lower-belief-level uncertainty to clarify their respective impacts on behavior and neural activity. Second, while the study identified cortical network associated with hierarchically uncertain decision-making the causal relationships among these networks remain unresolved. Functional connectivity analyses provided insight into network co-activation patterns but did not permit conclusions about directional interactions or hierarchical control within these systems. To advance mechanistic understanding, future studies should employ methodologies with higher temporal resolution such as magnetoencephalography (MEG) or intracranial electrophysiology, combined with causal inference techniques such as dynamic causal modeling or Granger causality analyses.

## Resource availability

### Lead contact

Further information and requests for resources should be directed to and will be fulfilled by the lead contact, Risa Katayama (katayama.risa.8d@kyoto-u.ac.jp).

### Material availability

This study did not generate new unique reagents.

### Data and code availability

#### Data

Anonymized behavioral data and de-identified imaging data are available on Open Science Framework: https://doi.org/10.17605/OSF.IO/WCFU7 and Zenodo: https://doi.org/10.5281/zenodo.15093166, respectively. Unthresholded group-level statistical maps are available from NeuroVault: https://neurovault.org/collections/FUAPHGRH/.

#### Codes

All codes supporting our main results presented in this study are available via the Open Science Framework (https://doi.org/10.17605/OSF.IO/WCFU7).

#### Additional information

Any additional information required to reanalyze the data reported in this paper is available from the lead contact upon request.

## Acknowledgments

This study was supported by a project (no. P20006) subsidized by the 10.13039/501100001863New Energy and Industrial Technology Development Organization (NEDO) and 10.13039/501100001691Japan Society for the Promotion of Science (JSPS) KAKENHI (nos. 22H04998 and 23H04676), Japan. S.I. was also partly supported by the 10.13039/100009619Japan Agency for Medical Research and Development (AMED) under grant no. JP23wm0625001. W.Y. was supported by MRC/Versus Arthritis (MR/W027593/1), United Kingdom, and the 10.13039/501100013373NIHR Oxford Health Biomedical Research Centre (views expressed are those of the authors and not necessarily those of the NIHR or the Department of Health and Social Care). R.K. was funded by the 10.13039/501100002241Japan Science and Technology Agency, the establishment of university fellowship toward the creation of science technology innovation (grant no. JPMJFS2123), Japan. K.A. was partly supported by 10.13039/501100001691JSPS KAKENHI (nos. 22H04998 and 24H02163), and 10.13039/100009619AMED (nos. 24gm6910012 and 24wm0625210). The funding agencies had no role in the study design, data collection and analysis, decision to publish, or manuscript preparation.

## Author contributions

S.I. conceived the project; R.K., W.Y., K.A., and S.I. designed the study; R.K. performed the experiments and analyzed the data; R.K. wrote the draft; and R.K., W.Y., K.A., and S.I. prepared the final manuscript.

## Declaration of interests

The authors declare no competing interests.

## Declaration of generative AI in scientific writing

During the preparation of this work the authors used ChatGPT (OpenAI) in order to assist with English translation and editing. After using this tool, the authors reviewed and edited the contents as needed and take full responsibility for the content of the published article.

## STAR★Methods

### Key resources table

**Table:** 

REAGENT or RESOURCE	SOURCE	IDENTIFIER
**Deposited data**		

Anonymized behavioral data	This paper	OSF: https://doi.org/10.17605/OSF.IO/WCFU7
Anonymized imaging data	This paper	Zenodo: https://doi.org/10.5281/zenodo.15093166
Unthresholded group-level statistical maps	This paper	NeuroVault: https://neurovault.org/collections/FUAPHGRH/

**Software and algorithms**		

Custom codes for behavioral analysis	This study	OSF: https://doi.org/10.17605/OSF.IO/WCFU7
Psychopy3	Peirce et al.	https://www.psychopy.org
MATLAB 2024a	The Mathworks, Natick, MA, USA	https://www.mathworks.com/products/matlab.html
SPM12	Wellcome Department of Cognitive Neurology, London, UK	https://www.fil.ion.ucl.ac.uk/spm/software/spm12/
R 4.2.0	R Core Team	https://www.r-project.org/
MRIcroGL	Chris Rorden	https://github.com/rordenlab/MRIcroGL/tree/master
TFCE Toolbox	S. Smith and T. Nichols	http://www.neuro.uni-jena.de/tfce/

### Experimental model and study participant details

#### Participants

Twenty-six healthy participants (13 females; aged 29.8 ± 9.5 [mean ± SD] years, age range 21–49 years) were recruited for the experiments and provided their written informed consent. This study was approved by the ethics committees of Advanced Telecommunications Research Institute International, Japan (Approval No. 157), and the Graduate School of Informatics, Kyoto University, Japan (KUIS-EAR-2022-010).

Prior to the functional magnetic resonance imaging (fMRI) scanning, all participants underwent a pre-experimental training task and engaged in a behavioral experiment outside the scanner the day before scanning session. All participants were assigned to the same experimental condition, as this was a within-subject design, and no group-level assignment or randomization was used. Two participants whose deck inference accuracy was below chance in the final phase of the task (the last quarter of the trials) were excluded from the analyses. In addition, two participants who chose the same confidence level in more than 75% of the trials were excluded from the imaging analyses. As a results, the final sample size were 24 participants for the behavioral analyses and 22 for the imaging analyses. The minimum number of participants was defined as 18, based on a power analysis (α = 0.05, 1−β = 0.8), with the effect size calculated from a previous study on hierarchical decision-making^4^ using G∗Power (http://www.gpower.hhu.de/). No exclusions were made based on participants’ sex or gender. As a result, the influence of sex, gender, or both on the results of this study was not assessed, potentially limiting the generalizability of our findings.

### Method details

#### Twenty-one card game task

The task was a two-choice task modeled after blackjack, where participants decided whether to draw an additional card with the aim of maximizing the reward by making their hands as high as possible without exceeding a total of 21. In each trial, participants were dealt with two cards, and had to choose whether to draw and take one additional card (hit) or keep their current hand (stay). The face-up card, with a score randomly selected between 8 and 18, was observable, while the face-down and additional card scores were hidden and drawn from decks with different probability distributions.

At the beginning of each game, the face-down and additional card decks were pseudo-randomly assigned and remained unchanged throughout the game. The face-down card could score either 1 or 4 points, drawn from one of two decks: Deck L (80% chance of drawing a 4-point card, 20% chance of a 1-point card; expected score 3.4) or Deck H (50% chance of drawing either score; 2.5), with Deck H introducing higher uncertainty and lower expected score. The characteristics of the face-down card deck, the probability of getting 1-point and 4-point cards, being used in the current game was presented in numerical values at the start of each game to the participants. This design allowed for separation of uncertainty regarding the face-down card and the additional card.

The additional card deck also consisted of two types: Deck 4 and Deck 6. These decks contained cards with scores ranging from 0 to 10 points, distributed according to differently biased binomial distributions: Deck 4, B (10,0.4), and Deck 6, B (10,0.6). The participants were not informed which deck was currently in use, but were informed of the possible decks and their probability distributions before the experiment.

The payoff for each trial was determined by the total score. If participants stayed, the total score was the sum of the face-up and face-down cards. If they hit, the additional card’s score was added. A total score of 21 or less resulted in a payoff equal to the score; if the score exceeded 21, the payoff was zero. Participants received monetary rewards based on their total payoff and the accuracy of their additional card deck inference in addition to a base monetary reward (5,375 ± 256.9 JPY).

During each trial, after the inter-trial interval (Step 1 in Figure 1B; 1–3 s), participants first reported their inference about the additional card deck (Step 2) and rated their confidence in using a 4-point scale (Step 3). After the interstimulus-interval (1–3 s), three cards were presented (Step 5; 4–6 s), and participants were instructed to decide to hit or stay during this time. Participants subsequently reported their decision (Step 6) and their confidence in the decision (Step 7). Afterward, the face-down card was flipped up, followed by the additional card subsequently (0.5 s after the face-down card disclosure, for 2 s). Even when the participants chose to stay, the score of the additional card was revealed. Each choice (deck inference, decision, and confidence levels) had a 3 s time limit to ensure prompt responses. Missed trials (0.92 ± 1.1%) were excluded from the analysis. Feedback for each choice was provided by highlighting the selected option with a white frame, without revealing whether it was correct.

The participants completed 28 games, each consisting of 16 trials: 20 games (320 trials) were conducted in the behavioral experiment outside the scanner, and 8 games (128 trials) took place during the scanning experiment. The behavioral experiment was conducted one day before the scanning experiment. The experiment followed a 2 (additional card deck) × 2 (face-down card deck) factorial design, with 5 games for each combination in the behavioral experiment and 2 games per combination in the scanning experiment.

#### Training task

Prior to the behavioral experiment, on the same day, the participants performed a training task outside the scanner to become familiar with the Twenty-one task. Unlike the main experiment, the training task explicitly indicated the deck type of the additional card (Deck 4 or 6) at the beginning of each game, eliminating the need for participants to report deck inference and confidence (Steps 2 and 3 in Figure 1B). The training task consisted of four games, one for each combination of the 2 [additional card deck] × 2 [face-down card deck] design, with each game consisting of 16 trials, for a total of 64 trials.

#### Behavioral model

To explain the participants’ behaviors in the Twenty-one task, we constructed a hierarchical information processing model. This model has two phases: deck inference phase, where participants update their evidence of the additional card deck and estimate the expected score based on the inference, and a decision phase to choose hit or stay based on the estimated value-belief using a soft-max function (Figure S3A). (Of note, we regarded the occurrence probability of the face-down scorecards as observable information; the deck type of the face-down card was indicated when the game started, and its expected score could be explicitly calculated).

In our experimental task design, the additional card was revealed at the end of each trial regardless of the participants’ decision; the deck inference in the *t*-th trial, therefore, was updated based on the history of the additional card scores up to the last trial, that is, the *(t-1)*-th trial. After the additional card for the *(t-1)*-th trial was revealed, the evidence for each corresponding deck candidate was updated, which allowed the participants to infer the deck type as of the higher evidence. The evidence update was determined by the normalized likelihood, provided by the latest additional card score; namely, the closer the score was to the minimum, 0, the more evidence would be added to the plausible deck (i.e., Deck 4). Conversely, when the score was closer to the maximum, 10, more evidence from Deck 6 would be accumulated. For simplicity, it was assumed that either evidence showing greater agreement with the latent additional card score was updated; that is, if the latest score was below four, only Deck 4 evidence was updated, whereas if it was above six, only Deck 6 evidence was updated. Because the score of the additional card drawn from Deck 4 or Deck 6 followed the binomial distribution B (10,0.4) or B (10,0.6), respectively (Figure 1A), the deck inference was processed using the following equations:

(Equation 1) $$\begin{array}{c} E_{t,D4}=\left\{ \begin{array}{cc} E_{t-1,D4}+\frac{P\left(A_{t-1}^{*}|\text{Deck}\;4\right)}{P\left(A_{t-1}^{*}|\text{Deck}\;4\right)+P\left(A_{t-1}^{*}|\text{Deck}\;6\right)} & \text{if}\;A_{t-1}^{*}<5\\ E_{t-1,D4} & \text{otherwise} \end{array}\right. \end{array}$$

(Equation 2) $$\begin{array}{c} E_{t,D6}=\left\{ \begin{array}{cc} E_{t-1,D6}+\frac{P\left(A_{t-1}^{*}|\text{Deck}\;6\right)}{P\left(A_{t-1}^{*}|\text{Deck}\;4\right)+P\left(A_{t-1}^{*}|\text{Deck}\;6\right)} & \text{if}\;A_{t-1}^{*}>5\\ E_{t-1,D6} & \text{otherwise} \end{array}\right. \end{array}$$

Here,

(Equation 3) $$\begin{array}{c} \left\{ \begin{array}{c} P\left(A_{t-1}^{*}|\text{Deck}\;4\right)=\frac{10!}{A_{t-1}^{*}!\left(10-A_{t-1}^{*}\right)!}0.4^{A_{t-1}^{*}}0.6^{\left(10-A_{t-1}^{*}\right)}\\ P\left(A_{t-1}^{*}|\text{Deck}\;6\right)=\frac{10!}{A_{t-1}^{*}!\left(10-A_{t-1}^{*}\right)!}0.6^{A_{t-1}^{*}}0.4^{\left(10-A_{t-1}^{*}\right)} \end{array}\right. \end{array}$$

(Equation 4) $$\begin{array}{ccc} P\left(d_{t}=\text{Deck}\;6\right)=\frac{1}{1+\exp\left\{-\beta_{k}\left[1,\Delta E_{t}\;\right]\right\}} & \text{where} & \Delta E_{t}=E_{t,D6}-E_{t,D4} \end{array}$$

where $A_{t-1}^{*}$ denotes the score of the additional card in the *(t-1)*-th trial. Although it is possible to assume that the additional card deck was inferred based on Bayesian optimal updating with a true posterior probability distribution, our proposed model showed better reproducibility of the participants’ deck inference behaviors than that (Figures S2A and S2D and Table S2; see also alternative models).

The deck confidence level, $c_{k}$, was correlated with the unsigned difference in the evidence (Figure S2B). Moreover, the deck confidence level was over-evaluated when participants’ current inference agreed with their previous inferences (Figure S2B). According to these results, deck confidence (between 0, the lowest, and 1, the highest) was defined based on the unsigned difference in evidence and the consistency rate of the deck inference in the game as follows (see also Figure S2C):

(Equation 5) $$\begin{array}{ccc} c_{k,t}=\frac{1}{1+\exp\left\{-\beta_{c_{k}}\left[1,\left|\Delta E_{t}\right|,\kappa_{t}\right]\right\}} & \text{where} & \begin{array}{cc} \kappa_{t}=\frac{1}{t}\sum_{l=1}^{t}\delta_{l}, & \delta_{l}=\left\{ \begin{array}{cc} 0 & if\;D_{l}\neq D_{t}\\ 1 & \text{otherwise} \end{array}\right. \end{array} \end{array}$$

$D_{t}$ indicates the deck type reported by the participants, and $\kappa_{t}$ represents the consistency rate of the participant’s deck inference.

Using the deck confidence level, as predicted by the proposed model (Equation 5), the additional card score was estimated as a weighted sum of the expected score from the inferred deck d ($E_{d}\left(A\right)$) and the expected score from the alternative deck $\bar{d}$ ($E_{\bar{d}}\left(A\right)$) as follows:

(Equation 6) $$\begin{array}{c} {\hat{A}}_{t}=\omega_{d_{t}}E_{d_{t}}\left(A\right)+\left(1-\omega_{d_{t}}\right)E_{\bar{d_{t}}}\left(A\right) \end{array}$$

where

(Equation 7) $$\begin{array}{c} \omega_{d_{t}}=\varepsilon -\gamma \left(1-c_{k,t}\right) \end{array}$$

When their deck inference was the most uncertain, the alternative deck type of deck inference was also taken into consideration with a certain probability, γ (Equations 6 and 7). It was also assumed that even when the participants reported the highest confidence in their deck inference, their deck inference were still influenced by the alternative deck with a probability of ε.

The decision was determined by the value-belief $V_{hit}$, that is, the difference between the busting threshold (=21) and the expected total score by choosing to hit, i.e., the sum of the face-up card score ($F_{u,t}^{*}$), expected score of the face-down card (${\hat{F}}_{d,t}$), and estimation of the additional card score (${\hat{A}}_{t}$). The face-down card score is calculated as $p_{F_{u}}^{*}+4\left(1-p_{F_{u}}^{*}\right)$ with the occurrence probability of the lower scorecard $p_{F_{u}}^{*}$ instructed to the participants in each game (see twenty-one card game task).

(Equation 8) $$\begin{array}{c} V_{hit,t}=21-\left(F_{u,t}^{*}+{\hat{F}}_{d,t}+{\hat{A}}_{t}\right) \end{array}$$

We used the value-belief given by Equation 8, rather than the difference between possible actions, because the total score resulting from choosing to hit will necessarily be higher than that from choosing to stay, unless it exceeds the busting threshold. The subjective utility for action selection is assumed to include not only $V_{hit}$ but also the interaction between $V_{hit}$ and $\left|V_{hit}\right|$. Therefore, in this model, the decision is formulated as follows:

(Equation 9) $$\begin{array}{c} P\left(a_{t}=\text{hit}\right)=\frac{1}{1+\exp\left\{-\beta_{i}\left[1,V_{hit,t},V_{hit,t}\left|V_{hit,t}\right|\right]\right\}} \end{array}$$

To modeling decision confidence level $c_{i}$, we introduce the decision simplicity, DS. In addition, according to the behavioral results indicating that the level of deck confidence also biased decision confidence, decision confidence was determined using the two-factorial utility as follows:

(Equation 10) $$\begin{array}{ccc} c_{i}=\frac{1}{1+\exp\left\{-\beta_{c_{i}}\left[{1,DS}_{t},c_{k,t}\right]\right\}} & \text{where} & {DS}_{t}=\left|\beta_{i}\left[1,V_{hit,t},V_{hit,t}\left|V_{hit,t}\right|\right]\right| \end{array}$$

To perform model fitting and validation, we also modeled the discretization of the deck- and decision confidence ($c_{k}$, $c_{i}$) into 4-point confidence reports with a noise parameter σ and a set of boundary thresholds $\theta =\left\{\theta_{c,1|2},\theta_{c,2|3},\theta_{c,3|4}\right\}$, as follows (Figure S2C):

(Equation 11) $$\begin{array}{c} \left\{ \begin{array}{c} \begin{array}{ccc} P\left(C_{t}=1\right) & = & \frac{\Phi \left(\theta_{c,1|2}-c\right)}{\sigma }\\ P\left(C_{t}=2\right) & = & \frac{\Phi \left(\theta_{c,2|3}-c\right)}{\sigma }-\frac{\Phi \left(\theta_{c,1|2}-c\right)}{\sigma } \end{array}\\ \begin{array}{ccc} P\left(C_{t}=3\right) & = & \frac{\Phi \left(\theta_{c,3|4}-c\right)}{\sigma }-\frac{\Phi \left(\theta_{c,2|3}-c\right)}{\sigma }\\ P\left(C_{t}=4\right) & = & 1-\frac{\Phi \left(\theta_{c,3|4}-c\right)}{\sigma } \end{array} \end{array}\right. \end{array}$$

Φ indicates the cumulative distribution function of standardized normal distribution. σ and θ were estimated for deck- and decision confidence separately. For simplicity, the second threshold ($\theta_{c,2|3}$) was set to be 0.5. The first and third thresholds were estimated for each participant.

#### Alternative models

To examine the reliability of the proposed deck-confidence-modulated decision model (DC-MD model), we prepared two alternative models. The first is a deck-confidence-independent decision model (DC-ID model; Figure S3A), which first infers the deck of the additional card based on the history of their scores, as in the DC-MD model, and then estimates the value-belief. However, this model assumes that participants rely solely on their inferred deck type when making decisions, independent of their confidence level in this inference, and that the deck confidence does not influence their decision confidence. Therefore, in this model, Equations 8 and 10 are replaced with:

(Equation 12) $$\begin{array}{c} V_{hit,t}=21-\left(F_{u,t}^{*}+{\hat{F}}_{d,t}+E_{d_{t}}\left(A\right)\right) \end{array}$$

(Equation 13) $$\begin{array}{c} c_{i}=\frac{1}{1+\exp\left\{-\beta_{c_{i}}\left[1,\;{DS}_{t}\right]\right\}} \end{array}$$

The second alternative model is a hierarchical Gaussian filtering model (HGF model; Figure S3D), which predict the score of the additional card incorporating.

(1) The lowest-level belief ($X_{1}$) which represents participants’ inferred deck-type ($X_{1}=0$ when interring the additional card deck-type as Deck 4, and $X_{1}=1$ when interring as Deck 6).
(2) The tendency to infer the deck-type as Deck 6 ($X_{2}$).
(3) The volatility of this tendency ($X_{3}$).

The input to this three-level HGF was defined by the normalized likelihood calculated from the latest additional card score ($A_{t-1}^{*}$).

(Equation 14) $$\begin{array}{c} l_{t}=\frac{P\left(A_{t-1}^{*}|\text{Deck}\;6\right)}{P\left(A_{t-1}^{*}|\text{Deck}\;4\right)+P\left(A_{t-1}^{*}|\text{Deck}\;6\right)} \end{array}$$

The posterior of the first-level belief $\mu_{1}$ was set to be equal to the input.

(Equation 15) $$\begin{array}{c} \mu_{1,t}=l_{t} \end{array}$$

The second-level posterior $\mu_{2}$ and its precision $\pi_{2}$ (inverse variance, $1/\sigma_{2}$) were updated as follows:

(Equation 16) $$\begin{array}{c} \mu_{2,t}={\hat{\mu }}_{2,t}+\sigma_{2,t}\delta_{1,t} \end{array}$$

(Equation 17) $$\begin{array}{c} \pi_{2,t}={\hat{\pi }}_{2,t}+{{\hat{\pi }}_{1,t}}^{-1} \end{array}$$

The prediction of $\mu_{2,t}$ (i.e., ${\hat{\mu }}_{2,t}$) is $\mu_{2,t-1}$. $\delta_{1,t}$ donates the first-level prediction error, formulated as $\mu_{1,t}-{\hat{\mu }}_{1,t}$. The variance of the first- and second-level prediction were given by the following:

(Equation 18) $$\begin{array}{ccc} {\hat{\sigma }}_{1,t}={\hat{\mu }}_{1,t}\left(1-{\hat{\mu }}_{1,t}\right) & \text{where} & {\hat{\mu }}_{1,t}=\text{sigmoid}\left({\hat{\mu }}_{2,t}\right) \end{array}$$

(Equation 19) $$\begin{array}{c} {\hat{\sigma }}_{2,t}=\sigma_{2,t-1}+\exp\left(\kappa \mu_{3.t-1}+\omega_{2}\right) \end{array}$$

Here, $\omega_{2}$ and κ denote the unweighted second-level learning rate and the coupling strength between the second- and third-level, respectively. $\mu_{3.t}$ represents the third-level belief posterior. The prediction of it (${\hat{\mu }}_{3,t}$) and its variance (${\hat{\sigma }}_{3,t}$) were defined as follows:

(Equation 20) $$\begin{array}{c} {\hat{\mu }}_{3,t}=\mu_{3.t-1} \end{array}$$

(Equation 21) $$\begin{array}{c} {\hat{\sigma }}_{3,t}=\sigma_{3,t-1}+\omega_{3} \end{array}$$

The third-level precision was updated by the following:

(Equation 22) $$\begin{array}{c} \pi_{3,t}={\hat{\pi }}_{3,t}+\frac{\kappa^{2}}{2}w_{2,t}\left(w_{2,t}+r_{2,t}\delta_{2,t}\right) \end{array}$$

$\omega_{3}$ represents the unweighted third-level learning rate. $\delta_{2}$, $w_{2}$ and $r_{2}$ denote the second-level prediction error, the weighting factor and the relative difference of environmental and information uncertainty, respectively, defined as follows:

(Equation 23) $$\begin{array}{c} \delta_{2,t}=\frac{\sigma_{2,t}+{\left(\mu_{2,t}-{\hat{\mu }}_{2,t}\right)}^{2}}{\exp\left(\kappa \mu_{3.t-1}+\omega_{2}\right)+\sigma_{2,t-1}}-1 \end{array}$$

(Equation 24) $$\begin{array}{c} w_{2,t}=\frac{\exp\left(\kappa \mu_{3.t-1}+\omega_{2}\right)}{\exp\left(\kappa \mu_{3.t-1}+\omega_{2}\right)+\sigma_{2,t-1}} \end{array}$$

(Equation 25) $$\begin{array}{c} r_{2,t}=2w_{2,t}-1=\frac{\exp\left(\kappa \mu_{3.t-1}+\omega_{2}\right)-\sigma_{2,t-1}}{\exp\left(\kappa \mu_{3.t-1}+\omega_{2}\right)+\sigma_{2,t-1}} \end{array}$$

The participants’ deck inference and deck confidence were processed using the following equations:

(Equation 26) $$\begin{array}{c} P\left(d_{t}=\text{Deck}\;6\right)={\hat{\mu }}_{1,t} \end{array}$$

(Equation 27) $$\begin{array}{c} c_{k,t}=\frac{1}{1+\exp\left\{-\beta_{c_{k}}\left[1,{\hat{\mu }}_{3,t}\right]\right\}} \end{array}$$

The model prediction of the participants’ expected additional card score ($\hat{A}$) was calculated as follows.

(Equation 28) $$\begin{array}{c} {\hat{A}}_{t}=\left(1-{\hat{\mu }}_{1,t}\right)E_{\text{Deck}\;4}\left(A\right)+{\hat{\mu }}_{1,t}E_{\text{Deck}\;6}\left(A\right) \end{array}$$

The value-belief and decision were formulated using the same equation as those of the DC-MD model (Equations 8 and 9). The utility of decision confidence level (Equation 10) was replaced with:

(Equation 29) $$\begin{array}{c} c_{i}=\frac{1}{1+\exp\left\{-\beta_{c_{i}}\left[{1,DS}_{t},{\hat{\mu }}_{3,t}\right]\right\}} \end{array}$$

For the deck inference phase, we also compared two alternative models, the averaging model and the optimal Bayesian model, with the proposed evidence-based updating model. In the averaging model, Equation 4 is replaced with:

(Equation 30) $$\begin{array}{ccc} P\left(d_{t}=\text{Deck}\;6|A_{t-1}^{*}\right)=\frac{1}{1+\exp\left\{-\beta_{k}\left[1,\Delta E_{t}\;\right]\right\}} & \text{where} & \Delta E_{t}=\frac{1}{t}\sum_{\tau =1}^{t}A_{\tau }^{*}-5 \end{array}$$

In the optimal Bayesian model, it is assumed that the inference of the additional card deck type is updated using an incremental Bayesian filtering process, and the deck confidence level is assessed based on the entropy of the deck inference as follows:

(Equation 31) $$\begin{array}{c} P\left(d_{t}|{A^{*}}_{t-1}^{1}\right)\propto P\left(A_{t-1}^{*}|d_{t-1}\right)P\left(d_{t-1}|{A^{*}}_{1}^{t-2}\right) \end{array}$$

(Equation 32) $$\begin{array}{ccc} c_{k}=\frac{1}{1+\exp\left\{-\beta_{c_{k}}\left[1,Q_{t},\kappa_{t}\right]\right\}} & \text{where} & Q_{t}=-\sum_{d_{t}}P\left(d_{t}|{A^{*}}_{1}^{t-1}\right)\log\left(P\left(d_{t}|{A^{*}}_{1}^{t-1}\right)\right) \end{array}$$

#### Model fitting and validation

In all the models, we simultaneously modeled the choices and confidence for deck inference and decision-making, respectively. The proposed model has 11 group-level and 8 individual-level parameters for the deck inference, and 13 group-level and 11 individual-level parameters for the decision-making. We place noninformative (or weakly informative) priors between sensible limits.

All group- and individual-level parameters were simultaneously estimated using Bayes’ rule by incorporating behavioral data (Table S1). To fit the models, three Markov chain Monte Carlo (MCMC) chains were run for 20,000 iterations after 10,000 burn-in samples were drawn with a thinning of 20, resulting in 3,000 posterior samples. The convergence of each estimated parameter was confirmed both visually via trace plots and statistically using the Gelman-Rubin test^43^; all parameters showed that $\hat{R}$ values were close to 1.0 (at most, smaller than 1.03 in this study), indicating sufficient convergence.

To validate the proposed DC-MD model, we computed the leave-one-out information criterion (LOOIC). We also used the agreement between the model’s prediction of the deck inference ($\hat{D}$), hit-or-stay decision ($\hat{a}$), corresponding confidence levels (deck confidence, ${\hat{C}}_{k}$; decision confidence, ${\hat{C}}_{i}$), and the actual reports by the participants ($D^{*}$, $a^{*}$, $C_{k}^{*}$ and $C_{i}^{*}$). These four types of states were predicted using the following equations.

(Equation 33) $$\begin{array}{c} {\hat{D}}_{t}={\mathrm{argmax}}_{d_{t}=\left\{\text{Deck}4,\text{Deck}6\right\}}P\left(d_{t}|{A^{*}}_{t-1}^{1}\right) \end{array}$$

(Equation 34) $$\begin{array}{c} {\hat{C}}_{k,t}={\mathrm{argmax}}_{C=\left[1,2,3,4\right]}\;P\left(C_{k,t}=C\right) \end{array}$$

(Equation 35) $$\begin{array}{c} {\hat{a}}_{t}={\mathrm{argmax}}_{a_{t}=\left\{\text{hit},\text{stay}\right\}}P\left(a_{t}\right) \end{array}$$

(Equation 36) $$\begin{array}{c} {\hat{C}}_{i,t}={\mathrm{argmax}}_{C=\left[1,2,3,4\right]}\;P\left(C_{i,t}=C\right) \end{array}$$

When Equations 33, 34, 35, and 36 yield multiple equally probable states (i.e., maximum a posteriori [MAP] estimates), we assume that the model randomly extracts one of the MAP estimates as its prediction. To avoid data circularity, we used data from 20 games in the behavioral experiment for parameter selection and data from 8 games in the scanning experiment for model validation.

We also performed a parameter recovery analysis to ensure that the model parameters were identifiable after model fitting. First, i) we randomly drew a set of the group-level parameters from the joint posterior distribution of the DC-MD model using the actual participants’ behaviors; namely, a group-level mean ($\mu_{\phi }$) and SD ($\sigma_{\phi }$) of the parameter ϕ. Then ii) the individual-level parameters $\phi_{i}$ for $N_{sim}$ synthetic participants were simulated by being randomly sampled from the corresponding group-level parameter with a normal distribution: $\phi_{i}∼N\left(\mu_{\phi },\sigma_{\phi }\right)$. We used $N_{sim}=50$. iii) Next, we simulated the behavioral data for the synthetic participants using the DC-MD model as a generative model; individuals’ deck inference, deck confidence level, hit-or-stay decision, and decision confidence level for 320 trials (i.e., 16 trials × 20 games) were sampled from the likelihood functions (Equations 4, 9, and 11) with $\phi_{i}$. iv) We subsequently fitted the DC-MD model to the simulated data with MCMC using JAGS, and obtained the posterior distribution of the parameters, followed by v) comparing whether the posterior distributions given the simulated data recovered the actual data generating parameters ($\phi_{i}$; Figure S5).

#### Image acquisition

A 3.0-Tesla Siemens MAGNETOM Prisma fit scanner (Siemens Healthineers, Erlangen, Germany) with a standard 64-channel phased-array head coil was used for image acquisition. We acquired interleaved T2∗-weighted echo-planar images (TR, 1000 ms; TE, 30 ms; flip angle, 50°; matrix size, 100 × 100; field of view, 200 × 200; voxel size, 2 × 2 × 2.5 mm; and number of slices, 66). Volume acquisition was synchronized with the onset of each delay period. We also acquired whole-brain high-resolution T1-weighted structural images using a standard MPRAGE sequence (TR, 2250 ms; TE, 3.06 ms; flip angle, 9°; field of view, 256 × 256; voxel size, 1 × 1 × 1 mm).

Imaging data were analyzed using SPM12 (Wellcome Department of Cognitive Neurology, London, UK). For each participant, all functional images were preprocessed, including slice-timing correction, spatial realignment, co-registration to the individual high-resolution anatomical image, normalization to an MNI template, and smoothing with a Gaussian kernel filter (FWHM, 8 mm). In addition, high-pass filtering with a cutoff of 128 s was applied to remove low-frequency drifts from the signal.

#### fMRI data analyses

Our standard GLM-based fMRI analyses employed event-related regressors convolved with canonical hemodynamic response function (HRF). The basic design matrix of the first-level analysis consisted of a constant term, 6 motion parameters produced during spatial realignment as nuisance regressors, and 9 event onset regressors (indicated by the event numbers in Figure 1B) in each session. All event-related regressors were modeled as delta functions. Parametric modulation analyses were conducted using the following GLMs, each of which included additional specific parametric modulators of interest (Figure S4A). The parametric modulators were orthogonalized in advance using the Gram-Schmidt method, rather than the built-in function in SPM.

GLM 1: The onset cue of the decision (Step 5 in Figure 1B) was modeled using two parametric modulators: (1) value-belief of the chosen action, that is, $V_{hit}$ when the participants chose to hit and ${-V}_{hit}$ otherwise, and (2) decision confidence level $c_{i}$ (Figure 4A and 4B). Both variables were calculated based on the computational model (DC-MD model), scaled to be in the range [0,1] and mean-centered. In this GLM, the decision confidence was orthogonalized with respect to the chosen value-belief, based on the assumption that the decision confidence is formed from the value-belief.

GLM 2: The onset cue of the decision was modeled using two parametric modulators: (1) deck confidence level $c_{k}$ and (2) chosen value-belief (same as GLM 1), calculated using the DC-MD model (Figure 4C). The variables were scaled to be in the range [0,1] and mean-centered. In this GLM, the chosen value-belief was orthogonalized with respect to the deck confidence, based on the assumption that the value-belief is modulated by the deck confidence level.

The aforementioned two-GLM approach focused on a clearer dissociation of cognitive processes at each stage of decision-making. To complement the prospective that these three variables of interest are integral to the decision-making process, we also constructed a GLM in which the onset cue of the decision was modeled using three parametric modulators, (1) deck confidence, (2) chosen value-belief, and (3) decision confidence, sequentially orthogonalized in this order (Figure S4D). However, due to a hierarchical relationship among these variables, simultaneously orthogonalizing and entering all three variables into a single GLM underestimated the effect of downstream variables as shared variance may be absorbed by earlier-stage regressors.

A random-effects analysis was performed at the group level using an anatomically localized cerebral cortex. Statistical thresholds were set at the voxel level of *p* < 0.001 (uncorrected) and cluster level of *p* < 0.05 (FWE-corrected). To further validate our findings, we conducted non-parametric permutation testing with threshold-free cluster enhancement (TFCE),^44^ as implemented in the TFCE Toolbox (http://www.neuro.uni-jena.de/tfce/). The results confirmed that the same brain regions exhibited significant effects (Figure S4E), supporting the robustness of our findings.

#### ROI-based activity time-course analysis

To test the time-series effect of the deck confidence level on BOLD activity in the ROIs, we first defined a participants-specific mask using a leave-one-subject-out procedure to ensure the statistical independence in the ROI analyses. For each participant, i) we defined an 8-mm sphere ROI (i.e., in the vIC, dACC, and preSMA) based on the group-level contrast re-estimated using the remaining *n*-1 participants. This sphere was centered at the local peak nearest to the peak MNI coordinates of the ROI, as identified in the aforementioned whole-brain GLM analyses (Table S3), within the re-estimated contrast. Then ii) we selected the supra-threshold voxels within this spherical ROI in the re-estimated contract for the subsequent ROI analysis. The pre-processed BOLD time courses were extracted by averaging the signals from these voxels within each ROI mask.

#### Functional connectivity analysis

We conducted psychophysiological interaction (PPI) analyses to evaluate how the activities in the vIC, dACC and preSMA related to the deck confidence level during decision-making were coupled with those in other regions of the brain. We used the 8-mm spherical ROI in the above three areas centered at the peak MNI coordinates from the parametric modulation contrast of the deck confidence (Figure 4C and Table S2; see also fMRI data analyses, GLM 2) as a seed region. Note that we used a peak coordinate of the local-maxima as the center coordinate for the dACC. To construct the PPI regressor, we first extracted the deconvolved physiological time series from the seed ROI using the SPM12 software. The deconvolved physiological time series were then multiplied by the psychological regressor (0–1 rescaled and mean-centered deck confidence level during the decision phase [5 s from the onset] in each trial) and further convolved by the HRF. The PPI regressor was also applied high-pass filtering with a cutoff of 128 s to remove low-frequency drifts and regressed out the variation due to head motion. To avoid confounding effects, we included the BOLD time series of the seed region and the convolved psychological regressor as nuisance variables in the GLM. The results of the first-level PPI regression for each participant were then applied to a random-effects group-level analysis using an anatomically localized cerebral cortex. Statistical thresholds were set at the voxel level of *p* < 0.001 (uncorrected) and cluster level of *p* < 0.05 (FWE-corrected).

### Quantification and statistical analysis

All behavioral data analyses were conducted using R 4.2.0. The stats, coin, and ARTool packages were used to perform statistical analysis. Specifically, the Wilcoxon signed rank test, Wilcoxon rank-sum test, and ANOVA with ART were used to compare conditions. Behavioral model fitting was performed within a Bayesian framework using JAGS in R with the runjags package.^45^ Details of the MCMC sampling settings and convergence diagnostics are provided in the STAR Methods section. For model comparisons, we used the loo packages to compute LOO-CV scores. Imaging data were analyzed using SPM12. ROI-based time-series analysis were conducted using general linear modeling, and multiple comparisons were corrected using the FDR correction (*p* < 0.05). All statistical tests were two-tailed unless otherwise noted. The statistical details (means, SEMs, statistical tests used, exact *p*-values) are reported in the results section and figure captions.
