# Supplementary material for: Neural mechanisms of confidence propagation in hierarchical partially observable decision-making
Source: iScience. 2025 May 29;28(7):112782. doi: 10.1016/j.isci.2025.112782 (PMC12205593; doi:10.1016/j.isci.2025.112782)
Supplement: Document S1. Figures S1–S5 and Tables S1–S3 [file mmc1.pdf]

iScience, Volume *28*

Supplemental information

Neural mechanisms of confidence propagation in hierarchical partially observable decision-making

Risa Katayama, Wako Yoshida, Ken-ichi Amemori, and Shin Ishii

Supplementary Figures

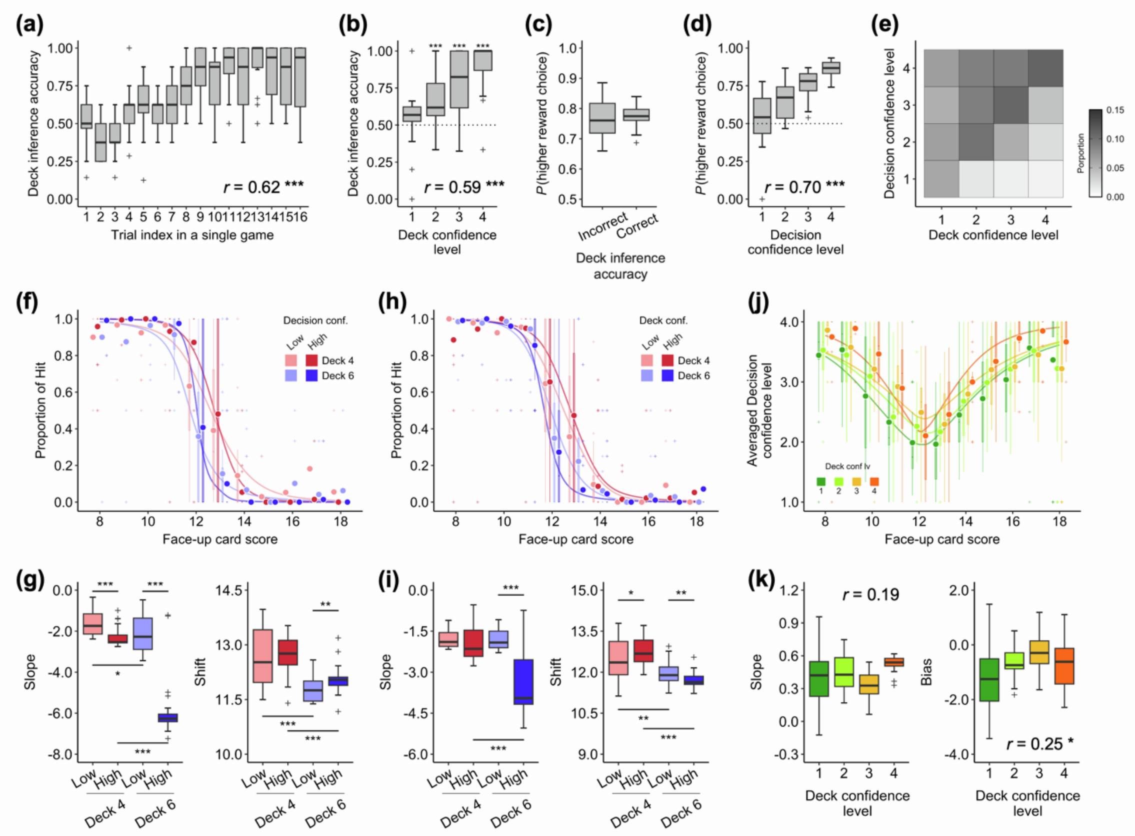

Figure S1. Behavioral results in the scanning experiment, related to Figure 1 and Figure 2.

(a) The estimation accuracy of the additional card deck type as a function of the index of trials within a single game. Accuracy increased progressively with the index of trial (r=0.624, p=6.8×10^{-43}). Each box extends from the lower to upper quartiles, with a horizontal line at the median. The whiskers indicate 1.5×Interquartile range (IQR), with cross markers denoting outliers. The dashed lines signify chance levels.

(b-k) The behavioral results analyzed using the same methods as in Figure 1c-f and Figure 2, revealed no significant differences between the behavioral and scanning experiments. This consistency indicates that the participant performance remained stable across experimental settings. Note that the sample size of the scanning experiment was considerably smaller (~120) than that of the behavioral experiment (Fig. 2, ~320).

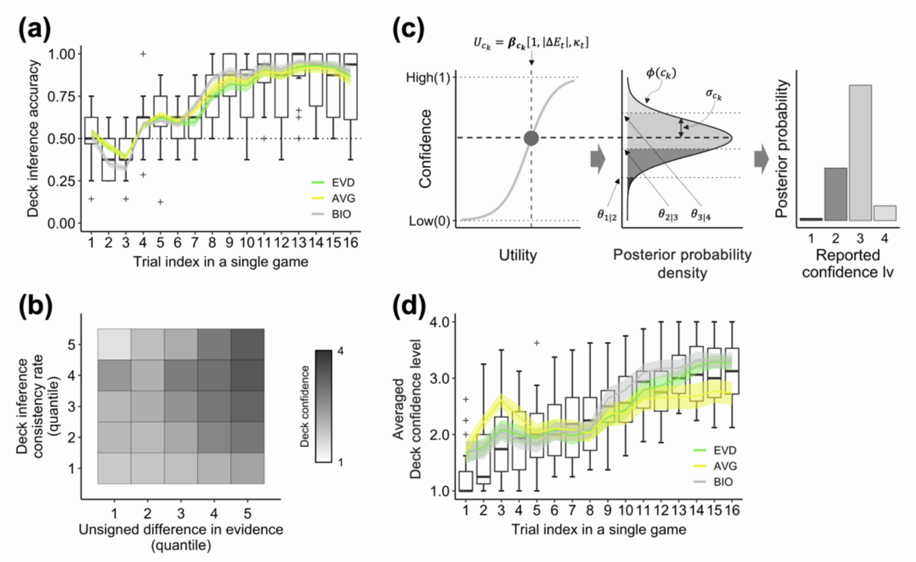

Figure S2. Model-based behavioral results of the deck inference, related to Figure 3.

(a) Comparisons of deck inference accuracy based on the participants' actual behavior (boxplot), based on estimation using the proposed evidence updating model (green), average updating model (yellow) and Bayesian ideal observer (gray; see **Methods** for details). The significant drop in inference accuracy on the second trial was solely due to the pseudo-random order of the additional card, and the Bayesian ideal observer also showed a similar drop in accuracy. Each box extends from the lower to upper quartiles, with a horizontal line at the median, the whiskers indicating 1.5×IQR and cross markers denoting outliers. Solid lines with shaded areas indicate the mean ± SEM. Each data point represents the average accuracy for an individual participant.

(b) Averaged deck confidence level as a function of the unsigned difference in evidence and consistency rate of participants' deck inference, i.e., the agreement of the current deck inference with the previous ones in the game. The data were binned according to the quintiles of each variable. Each square within the heatmap represents the average deck confidence level across the participants.

(c) Model of the subjective deck confidence. The deck confidence was predicted from the logistic regression model with the unsigned difference in evidence and the consistency rate of the deck inference (c_k; low [0] to high [1]; left panel). The participant's deck confidence report was assumed to be drawn from a probability distribution, which was calculated based on a Gaussian distribution centered on c_k and the set of thresholds $\boldsymbol{\theta}$ (lower panels). The set of beta weights and thresholds was fitted to each participant (see **Methods** for details). $\phi(x)$ indicate the probability density function of the standard normal distribution.

(d) Comparisons of the deck confidence levels based on the participants' actual behavior (boxplot), based on the estimation using the proposed evidence updating model (green), average updating model (yellow)

and Bayesian ideal observer (gray; see **Methods** for details). Each box extends from the lower to upper quartiles, with a horizontal line at the median, the whiskers indicating 1.5×IQR and cross markers denoting outliers. Solid lines with shaded areas indicate the mean ± SEM. Each data point represents the average confidence level for an individual participant.

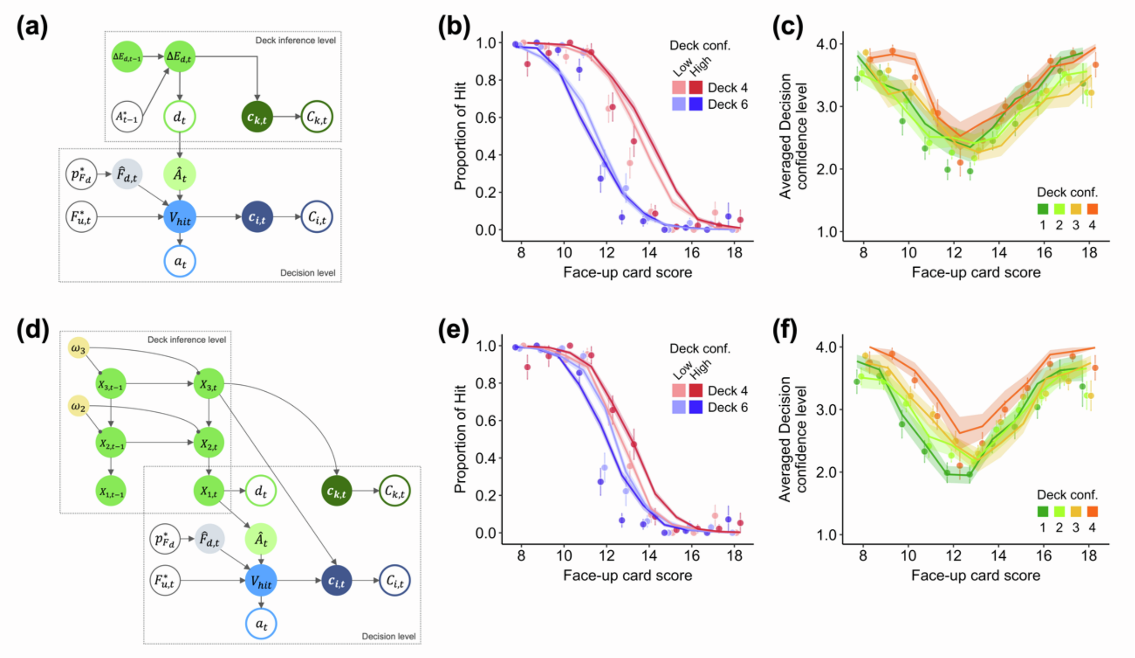

Figure S3. Behavioral analysis results based on the alternative information processing models, related to Figure 3.

(a,d) Schematic description of the alternative models; the deck-confidence-independent decision (DC-ID) model (a), and the hierarchical-Gaussian-filtering (HGF) model (d). The details of the alternatives are described in the Methods.

(b-c, e-f) The behavioral analysis results using the DC-ID model (b-c) and the HGF model (e-f). The analysis methods were the same as those shown in Figure 3.

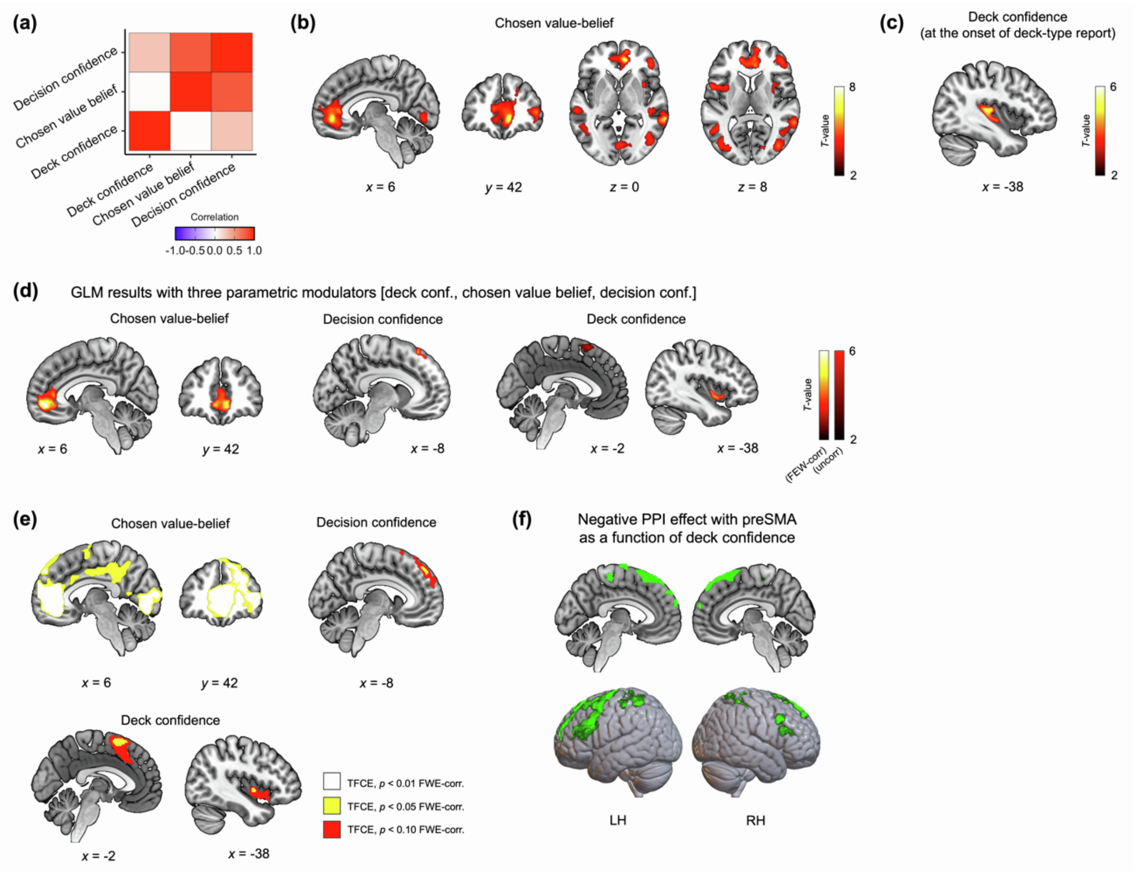

Figure S4. Supplementary results of the parametric imaging analyses and PPI analysis, related to Figure 4 and Figure 5.

(a) Correlation matrix of decision-making-related variables derived from the DC-MD model.

(b) Neural representation of the chosen value belief at the onset of the decision-making (Step 5 in Figure 1b).

(c) Brain activity correlating deck confidence level at the onset of the deck inference report (Step 2 in Figure 1b).

(d) Brain regions exhibiting the significant effects of the deck confidence, chosen value-belief and decision confidence at the onset of decision-making. These three variables were included in a single GLM, serially orthogonalized in this order.

(e) Brain regions exhibiting significant effects of the chosen value-belief, decision confidence, and deck confidence at the onset of decision-making, identified using TFCE correction.

(f) Brain regions that show a decrease in functional connectivity with the preSMA as a function of the deck confidence level during the decision (for 5 s from the onset of decision).

In the above figures, the clusters are significant at p<0.05 FWE-corrected, with the cluster-defining threshold p<0.001 uncorrected.

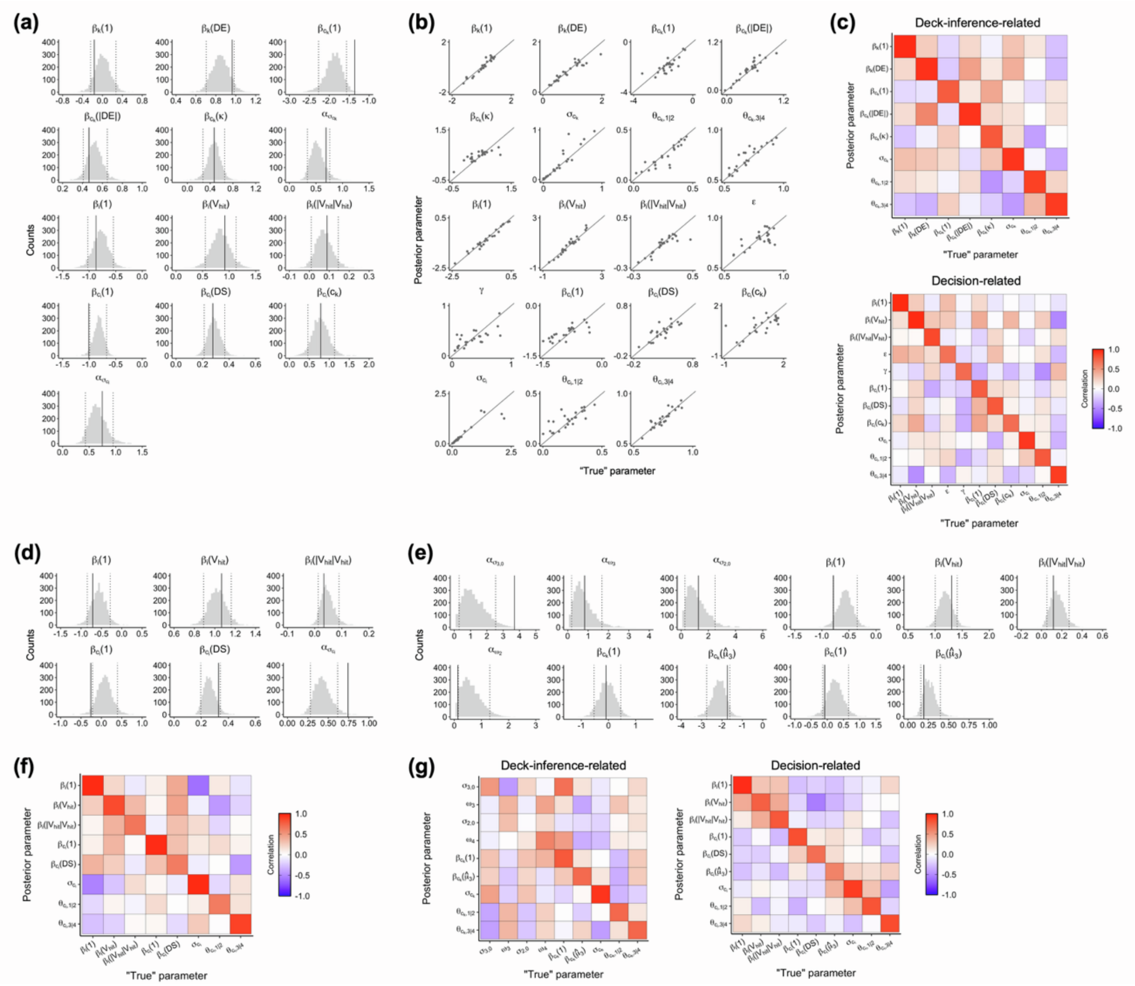

Figure S5. Parameter recovery results, related to STAR Methods.

(a) A random sample of group-level parameters was drawn from the posterior distribution of the DC-MD model (Supplementary Table 1) to simulate 24 synthetic participants' behavioral data (deck inference, deck confidence level, hit-or-stay decision, and decision confidence level) for the Twenty-one task. The generative model was then fitted to the generated data from these synthetic participants, after which the parameter estimates were compared with the data-generating parameters. For the group-level, the data-generating parameters (i.e., "true" parameters, vertical line) fell within 95% HDI of each parameter's posterior density (dotted lines).

(b) For the individual-level, the data-generating parameters and the estimated posterior parameters were correlated along the identity line.

(c) Correlation matrices of the deck-inference-related (left) and decision-related parameters (right), which were used for the data generation and estimated as the posterior parameters.

(d-f) Parameter recovery results for the alternative models. For the group-level, the data-generating parameters (i.e., “true” parameters, vertical lines) fell within 95% highest density interval (HDI) of each parameter’s posterior density (dotted lines) from the deck-confidence-independent decision (DC-ID) model (d). Correlation matrices of the DC-ID model parameters which were used for the data generation and their corresponding estimated posterior parameters (f). (e,g) show the recovery results for the hierarchical Gaussian filtering (HGF) model. For details on these alternative models, please refer to the **Alternative models.**

Supplementary Tables

Table S1. Estimated parameters using the deck-confidence-modulated decision model, related to Figure 3. This table summarizes the group-level posterior estimates (mean and 95% highest density interval [HDI]), the individual-level posterior estimates (participant-wise mean and SD), and describes the role of each parameter within the model.

	**Name**	**Explanation**	**Group-level posterior (mean [95% HDI])**	**Individual-level posterior (mean ± SD)**
Deck inference	$\beta_k(1)$	Bias toward inferring as "deck 6"	-0.25 [-0.27, 0.22]	-0.026±0.54
	$\beta_k(\Delta E)$	Effect of difference in evidence	1.00 [0.77, 1.24]	1.00±0.50
Deck confidence	$\beta_{c_k}(1)$	Deck confidence bias	-1.31 [-1.78, -0.84]	-1.30±0.89
	$\beta_{c_k}(\|\Delta E\|)$	Effect of unsigned difference in evidence	0.60 [0.42, 0.80]	0.59±0.36
	$\beta_{c_k}(\kappa)$	Effect of deck inference consistency rate	0.57 [0.31, 0.91]	0.56±0.53
	σ_{c_k}	Discretization noise for deck confidence report	-	0.22±0.067
	$\theta_{c_k,1\|2}$	Boundary threshold of deck confidence report (level 1/2)	-	0.25±0.10
	$\theta_{c_k,3\|4}$	Boundary threshold of deck confidence report (level 3/4)	-	0.73±0.091
Decision	$\beta_i(1)$	Bias toward choosing to hit	-0.72 [-1.04, -0.40]	-0.71±0.70

	$\beta_k(V_{hit})$	Effect of value-belief	1.28 [1.10, 1.47]	1.28±0.27
	$\beta_k(V_{hit} \vert V_{hit} \vert)$	Effect of unsigned value-belief as a modulator of (signed) value-belief	0.075 [0.016, 0.14]	0.071±0.088
	γ	Deck confidence weighting	-	0.28±0.093
	ε	Baseline effect of alternative deck	-	0.81±0.086
Decision confidence	$\beta_{c_i}(1)$	Decision confidence bias	-0.87 [-1.12, -0.63]	-0.86±0.36
	$\beta_{c_i}(DS)$	Effect of decision simplicity	0.27 [0.20, 0.34]	0.27±0.11
	$\beta_{c_i}(c_k)$	Deck confidence bias effect	0.91 [0.54, 1.25]	0.93±0.55
	σ_{c_i}	Discretization noise for decision confidence report	-	0.20±0.072
	$\theta_{c_i,1\vert 2}$	Boundary threshold of decision confidence report (level 1/2)	-	0.24±0.10
	$\theta_{c_i,3\vert 4}$	Boundary threshold of decision confidence report (level 3/4)	-	0.73±0.81

Table S2. Bayesian model comparison results, related to Figure 3. This table summarizes the validation performance of each model, including the leave-one-out information criteria (LOOIC) with its standard error, model weight (calculated using the Bayesian bootstrap, where a higher value indicates better prediction performance), and ΔELPD (the difference in the expected log pointwise predictive density relative [ELPD] to the best-performing model, along with its standard error). Additionally, the table reports the number of free parameters at the individual-level (No. of param). Model abbreviations: DC-MD, deck-confidence-modulated

decision model; DC-ID, decision-confidence-independent decision model; HGF, hierarchical Gaussian filtering model; EVD, evidence updating model; AVG, average updating model; BIO, Bayesian ideal observer model.

**Model**	**LOOIC (SE)**				**Weight**	**ΔELPD**	**No. of param**
	Deck inference	Deck confidence	Decision	Decision confidence			
DC-MD (with EVD)	2919.6 (73.8)	6578.9 (99.3)	1661.0 (83.5)	6754.0 (151.8)	0.97	0	19
DC-ID (with EVD)	2919.6 (73.8)	6578.9 (99.3)	1834.7 (76.5)	7099.1 (168.1)	0.00	-261.1 (35.0)	16
HGF	3473.5 (24.1)	6362.0 (93.1)	1679.5 (80.2)	6601.1 (144.8)	0.03	-101.6 (49.2)	18
Deck inference model							
EVD	2919.6 (73.8)	6578.9 (99.3)	-	-	0.999	0	8
AVG	2706.8 (59.6)	7042.9 (84.3)	-	-	0.001	-125.5 (34.7)	8
BIO	3821.8 (30.1)	6331.7 (88.1)	-	-	0	-327.4 (42.2)	8

Table S3. Peak voxels of the region where evoked activity was positively correlated with the chosen value belief, the decision confidence level, and the deck confidence at the onset of the decision, related to Figure 4.

Regions	L/R	MNI coordinates			z-value	No. of voxels
Chosen value-belief						
Pregenual cingulate cortex	L/R	8	44	-2	5.63	1076
Anterior prefrontal cortex	R	44	40	4	4.28	587
Dorsal anterior insular cortex	L	-32	4	14	4.49	366
Dorsal anterior insular cortex	R	30	2	14	4.16	204

Middle temporal gyrus	R	62	-40	-2	4.99	4301
Middle occipital gyrus	R	-32	-78	12	4.49	1037
Superior temporal gyrus	L	-58	-28	2	3.92	238
Decision confidence						
Dorsomedial prefrontal cortex	L/R	-8	42	46	4.57	624
Deck confidence						
Ventral insular cortex	L	-40	-2	0	4.50	333
Presupplementary motor area	L/R	-4	18	60	3.90	355
